# Human Y Chromosome Base-Substitution Mutation Rate Measured by Direct Sequencing in a Deep-Rooting Pedigree

**DOI:** 10.1016/j.cub.2009.07.032

**Published:** 2009-09-15

**Authors:** Yali Xue, Qiuju Wang, Quan Long, Bee Ling Ng, Harold Swerdlow, John Burton, Carl Skuce, Ruth Taylor, Zahra Abdellah, Yali Zhao, Asan, Daniel G. MacArthur, Michael A. Quail, Nigel P. Carter, Huanming Yang, Chris Tyler-Smith

**Affiliations:** 1 The Wellcome Trust Sanger Institute, Hinxton, Cambs CB10 1SA, UK; 2 Department of Otorhinolaryngology-Head and Neck Surgery and Institute of Otolaryngology, Chinese People's Liberation Army General Hospital, 28 Fuxing Road, Beijing 100853, China; 3 Beijing Genomics Institute at Shenzhen, Shenzhen 518000, China

**Keywords:** EVO_ECOL

## Abstract

Understanding the key process of human mutation is important for many aspects of medical genetics and human evolution. In the past, estimates of mutation rates have generally been inferred from phenotypic observations or comparisons of homologous sequences among closely related species [1–3]. Here, we apply new sequencing technology to measure directly one mutation rate, that of base substitutions on the human Y chromosome. The Y chromosomes of two individuals separated by 13 generations were flow sorted and sequenced by Illumina (Solexa) paired-end sequencing to an average depth of 11× or 20×, respectively [4]. Candidate mutations were further examined by capillary sequencing in cell-line and blood DNA from the donors and additional family members. Twelve mutations were confirmed in ∼10.15 Mb; eight of these had occurred in vitro and four in vivo. The latter could be placed in different positions on the pedigree and led to a mutation-rate measurement of 3.0 × 10^−8^ mutations/nucleotide/generation (95% CI: 8.9 × 10^−9^–7.0 × 10^−8^), consistent with estimates of 2.3 × 10^−8^–6.3 × 10^−8^ mutations/nucleotide/generation for the same Y-chromosomal region from published human-chimpanzee comparisons [5] depending on the generation and split times assumed.

## Results

The sequences of two Y chromosomes 13 generations apart on the same pedigree are expected to be identical outside the pseudoautosomal regions, except for mutations that have occurred during these generations. We investigated a Chinese family carrying the DFNY1 Y-linked hearing-impairment mutation [6] and genotyped two family members, DFNY1-66 (affected) and DFNY1-101 (unaffected), who were separated by 13 generations, with 67 Y-STRs [7]. We found that their Y haplotypes were indeed identical at all these loci (Table S1 in the Supplemental Data). We then sequenced the flow-sorted Y chromosomes to high depth and searched for base-substitution differences between them. To do this, we aligned the reads to the Y chromosome reference sequence by using the program MAQ with its default settings, identified positions that differed between the reference and each of the sequenced Y chromosomes (“default SNPs”), and compared the two lists of default SNPs. In this initial comparison we identified 12,909 DFNY1-66-specific SNPs and 22,865 DFNY1-101-specific SNPs, far more than expected; indeed, examination of the data suggested that the vast majority of default SNPs represented base-calling or alignment errors. We then developed a strategy to identify the small minority of genuine mutations.

### Identification of Candidate Mutations

For this, we made use of “gold standard” SNPs validated and placed on the Y-chromosomal phylogeny by the Y Chromosome Consortium (YCC) [8]. Except for the *AZFa* region 12,838,588–13,879,980, which originates from a haplogroup G individual, and some small gaps [9], the reference sequence is derived largely from a haplogroup R1b individual, whereas the DFNY1 Y chromosomes fell into haplogroup O3a. On the basis of the YCC phylogeny, 54 positions are expected to differ between the R1b section of the reference sequence and an O3a chromosome. After excluding one indel (M175) and three SNPs that lay in repeated regions and correcting one YCC typographical error (M269), we found all of the expected SNPs in our default SNP lists. We therefore reasoned that our SNP lists contained true Y-chromosomal variants, possibly including new mutations, but also a vast excess of false-positive calls and that we needed to identify suitable criteria for distinguishing between true and false calls. To find these criteria, we determined the MAQ parameters measuring the quality of base calling, read mapping, and coverage associated with these gold-standard SNPs (Table 1) and used them to filter the entire default SNP sets. This procedure resulted in a much shorter list of 18 first-class SNPs; relaxing the criteria slightly added another five second-class ones, yielding in all 23 candidates (10 from DFNY1-66 and 13 from DFNY1-101; Table 2).

### Verification of the Candidate Mutations by Capillary Sequencing

**Figure 1:**
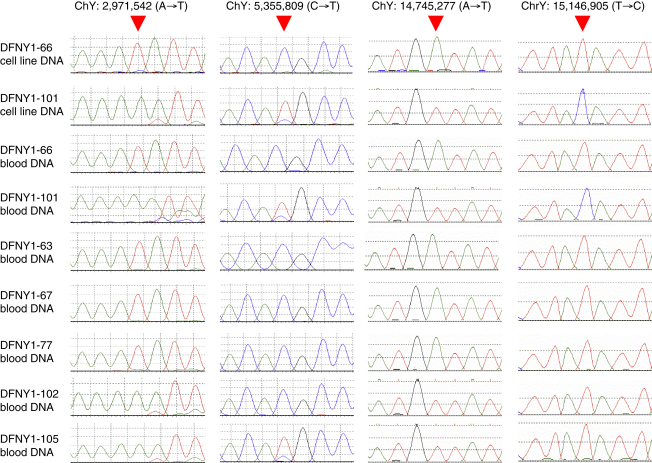
Capillary Sequence Traces of the Four Confirmed Mutations in Cell Line and Blood DNAs The red arrowhead indicates the mutant position.

**Figure 2:**
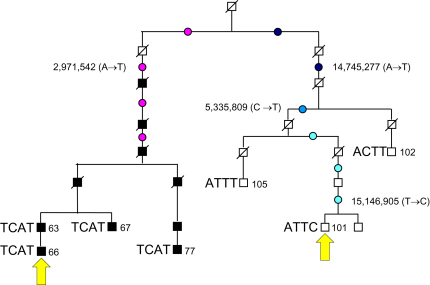
Locations of the Four Confirmed Mutations on the DFNY1 Pedigree The sequenced chromosomes (66 and 101) are indicated by yellow arrows, and the additional family members typed for mutations are labeled with their numbers and haplotypes at the mutant positions. The possible locations of each mutation are shown by one or more colored circle. Filled squares represent DFNY1 affected individuals; open squares represent unaffected individuals.

We next amplified the region spanning each candidate mutation from each of the cell lines used for chromosome sorting and sequenced them by conventional capillary sequencing. Twelve out of 18 (67%) first-class candidate mutations were confirmed in the cell-line DNA, and 0/5 (0%) second-class candidate mutations were confirmed. It therefore appears that the filtering criteria used were highly effective in identifying true mutations and that no more would be discovered if these criteria were relaxed. Nevertheless, mutations in cell-line DNA represent a combination of germline mutations carried by the donor and somatic mutations that have accumulated subsequently in culture [10, 11]. Blood DNA was available from both donors and from additional family members and was examined by capillary sequencing. Only four of the mutations were present in blood DNA (33%; Figure 1). Analysis of blood DNA from additional family members verified that three of the four mutations were also transmitted in the family and that the mutations had all occurred at different positions in the pedigree (Figure 2).

### Estimation of Mutation Rate

In order to estimate the mutation rate, we need to know, in addition to the number of mutations, the length of the region contributing data and the number of years or generations separating the chromosomes. Although the euchromatic male-specific region is ∼24 Mb in length, we excluded gaps in the reference sequence, highly repeated sections, and palindromes from our analysis, and we also required adequate coverage in both individuals. Applying the same filtering criteria for nonmutant positions as for candidate mutations, including a minimal coverage of three reads (Table 1), yielded ∼10.15 Mb of DNA. The chromosomes were separated by 13 generations, and the common ancestor of the two individuals was born in approximately 1805. The mutation rate is therefore 1.0 × 10^−9^ mutations/nucleotide/year (95% CI: 3.0 × 10^−10^–2.5 × 10^−9^), or 3.0 × 10^−8^ mutations/nucleotide/generation (95% CI: 8.9 × 10^−9^–7.0 × 10^−8^). This rate is consistent with estimates derived from published human-chimpanzee Y chromosome comparisons [5, 9] of the same ∼10.15 Mb region at 1.5 × 10^−9^-2.1 × 10^−9^ mutations/nucleotide/year for split times of 5-7 million years and 2.3 × 10^−8^–6.3 × 10^−8^ mutations/nucleotide/generation if a generation-time uncertainty of 15–30 years is included.

### Mutation Type

Substitutions between different bases occur at different rates. Among the four mutations we identified, two were A>T mutations, one was a C>T (from a CpG dinucleotide), and one was a T>C mutation. Comparisons of the same ∼10.15 Mb region in human and chimpanzee Y chromosomes identified 21,278 A-T/234,420 total mutations, and comparisons of variants between the reference sequence and all filtered SNPs in the individual we sequenced identified 46 A-T/629 total mutations. The enrichment of A-T mutations is marginally significant (p = 0.04 for the comparison of these observed A-T mutations with human-chimpanzee differences and p = 0.03 for the comparison with human polymorphisms, Fisher exact test) and merits re-examination when more human Y mutations are identified. In contrast, the somatic mutations did not differ from expectation.

## Discussion

Human mutation rates are important for understanding many aspects of evolution and medicine, and attempts to estimate them date back to Haldane's prescient 1935 figure of 2 × 10^−5^ mutations/gene/generation for the haemophilia gene [1]. This rate is equivalent to 2 × 10^−8^ mutations/nucleotide/generation if mutations at 1,000 nucleotides could generate haemophilia. Similarly, Kondrashov's estimate at 20 loci causing Mendelian disorders was 1.8 × 10^−8^ mutations/nucleotide/generation [2]. Alternative estimates for human and chimpanzee sequences that are likely to be neutral have also been similar: for example ∼2.5 × 10^−8^ mutations/nucleotide/generation [3]. Yet the mutation rate depends on local context; it varies over a scale that ranges from pairs of nucleotides (e.g., CpG dinucleotides show an approximately 10× higher rate of base substitution than the average) to entire chromosomes (e.g., the Y chromosome shows a rate several times higher than autosomes because of its restriction to the male germ line, where more cell divisions occur per meiosis) [12]. It has not previously been possible to measure base-substitution mutation rates directly by sequencing human nuclear DNA in families, but this has been done for the mtDNA HVSI, where a controversy has emerged over whether the “pedigree rate” measured in family studies is consistent with the “evolutionary rate” inferred from comparisons of different species or whether it is substantially faster [13]. The ability to measure nuclear rates directly, offered by advances in sequencing technology, now promises additional insights into these areas.

Current next-generation sequencing technologies such as the Illumina platform used here have a high base-calling error rate, perhaps 1%, and have the additional feature that the short reads obtained need to be mapped to the reference sequence; this feature is potentially error prone for non-unique sequences. We overcame base-calling errors by using high-quality calls and high coverage (mean 11× and 20×, respectively) and avoided mapping errors by excluding the extensive duplicated (“palindromic”) and highly repeated sections of the reference sequence from the analysis, as well as applying the filtering criteria listed in Table 1. We then tested all candidate mutations by capillary sequencing, and thus we are confident that the false-positive rate in the final dataset is effectively zero. The false-negative rate is more difficult to measure, but three lines of reasoning suggest that it is low. First, relaxing the candidate-mutation filters to include second-class candidates did not identify any additional mutations (Table 2). Second, in the capillary verification experiments, about 20 kb was sequenced from both chromosomes, and no unexpected mutations were discovered. Third, all of the expected gold-standard YCC SNPs were detected. Because these are detected by comparison with the reference sequence in the same way as mutations, we can use this measurement to estimate a false-negative rate of <2% at the positions that differ between the DFNY1 and reference sequences. Thus, we conclude that the measured rate is reliable.

In the current study, two DFNY1-family Y chromosomes separated by 13 generations were resequenced. Because one carries the DFNY1 mutation and the other does not, the question arises as to whether the mutations detected might relate to the DFNY1 phenotype rather than representing the neutral rate. Three of the four can be eliminated as causal because they do not segregate with the phenotype. The fourth (ChrY: 2,971,542 A>T) segregates with the phenotype but lies in a region devoid of genes and seems unlikely to be causal because a compelling candidate mutation—a rearrangement located outside the ∼10.15 Mb region scanned here—has been identified (our unpublished data). We therefore conclude that the SNP mutations observed do indeed represent the neutral rate.

The measured mutation rate has wide confidence intervals, but in the future these could be narrowed substantially if more sets of related males were sequenced, and they could in principle be more precise than rates inferred from comparisons of related species, which are limited by uncertainties in the fossil record and the generation times of extinct ancestors. No discrepancy in the pedigree or evolutionary rate was evident. Although mutations in cell culture are expected, the contrast between 8/8 mutations in one cell line and 0/8 in the other was not (p = 0.008) and suggests the influence of unknown mutagenic environmental factors or, more likely, a mutagenic genotype specific to DFNY1-101, and it illustrates how different somatic mutation rates can be in related cell lines. Two of the cell line mutations (4,633,474 C>T and 4,980,623 T>G; Figure S1) were mixtures of ancestral and mutant alleles, but the other six were fixed (3,957,219 G>A, 4,939,256 T>C, 12,063,011 C>G, 15,126,873 T>C, 20,627,064 C>G, and 27,095,961 A>G).

In conclusion, we have shown that one can use next-generation sequencing technology to measure the very low mutation rate of human nuclear DNA reliably. The mutation rate observed is consistent with that inferred from evolutionary comparisons but can potentially be measured more precisely and provide new insights into human mutation processes.

## Experimental Procedures

### Data Generation

This study was approved by the sample donors and by the Committee of Medical Ethics of the Chinese PLA General Hospital. Lymphoblastoid cell lines from two members of the DFNY1 family [6], DFNY1-66 and DFNY1-101 separated by 13 generations, were established. Flow sorting of Y chromosomes by standard procedures [14, 15] provided ∼520 ng DNA from DFNY1-66 and ∼640 ng from DFNY1-101. Paired-end libraries of ∼200 bp fragments were constructed, and 35 bp from each end were sequenced with Illumina (Solexa) technology [4]. After quality control and removal of duplicate reads, 11× and 20× mapped coverage of the Y reference sequence was obtained from DFNY1-66 and DFNY1-101, respectively. Mapping and SNP calling (identifying positions that differed from the reference sequence) were carried out with MAQ [16].

### Data Filtering to Identify Candidate Mutations

We set up the filter parameters by using the gold-standard Y-SNPs expected from the well-established Y-chromosomal phylogeny [8]. We determined the haplogroup for the two DFNY1 individuals to be O3a1 by typing a standard set of Y-SNPs including M122, whereas the haplogroup of most of the reference sequence is R1b (the non-R1b section was excluded from this part of the analysis). In total, 54 SNPs separate these two haplogroups according the current Y-chromosomal phylogeny [8], but three of these lie in duplicated regions and were excluded from our analysis along with one indel, which would not be detected with our settings. We therefore expected to see 50 of these SNPs in both samples. They were present, along with many other SNP calls in the default MAQ SNP files. We therefore set up the filtering parameters on the basis of the 50 SNPs. Five parameters can be used from the MAQ SNP file: consensus quality, read depth (coverage), the average number of genomic hits of reads covering this position (uniqueness), the highest mapping quality of the reads covering this position (mapping quality), and the quality difference between the major allele and the minor allele (SNP scores). We used different values for the two samples because the coverage and data quality differed between the samples. We also did not allow heterozygous calls, leading to the settings listed in Table 1. We applied these filters to the MAQ default outputs and identified all SNPs specific for each individual to create a filtered list of candidate mutations (Table 2). To define the second-class candidates, we relaxed the parameters for DFNY1-66 candidate SNP calling to include uniqueness less than 5 and coverage more than 1×.

### Verification by ABI Capillary Sequencing

For the filtered candidate mutations, we designed PCR primers by using Primer3 [17] (http://frodo.wi.mit.edu/) to amplify 400–700 bp fragments (primer sequences and PCR conditions are in Table S2), purified them by standard ExoSAP treatment, and sequenced them by using BigDye terminator chemistry on both forward and reverse strands [18]. Initial analyses were performed on the cell line DNAs from the two individuals. Candidate mutations confirmed in the cell-line DNAs were then sequenced in blood DNAs from the same individuals as well as five other family members (Figure 1). All the confirmed candidate mutations are supported by four or more capillary sequence reads.

### Mutation-Rate Calculations

The total length of sequence investigated was determined from the number of mapped bases that met the same criteria in terms of coverage as the candidate mutation filter; this number was 3–14 for DFNY1-101 and 3–20 for DFNY1-66 when the palindrome regions, high repeats, and gaps described in [9] were excluded. The regions included are shown in Documents S1 and S2 (.wig), which can be uploaded to the UCSC browser (http://genome.ucsc.edu/cgi-bin/hgGateway) for detailed viewing. We calculated the mean mutation rate and 95% confidence interval as described [19].

## Figures and Tables

**Table 1:** Parameters for Filtering Candidate Mutations from the MAQ SNP Calls

Parameter	DFNY1-66	DFNY1-101
Mapping quality	63	63
Consensus quality	>28	>35
Coverage	3∼20	3∼14
SNP scores	>28	>30
Uniqueness	1.00	1.00
No heterozygote call	TRUE	TRUE
Good call in the other sample	TRUE	TRUE

**Table 2:** Details of the Filtered Candidate Mutations

Chromosome Coordinate	Base	DFNY1_101 Pileup		DFNY1_66 Pileup		Confirmation	
		Coverage	Calls^1^	Coverage	Calls^1^	Cell-Line DNA	Blood DNA
First Class							

chrY:3,957,219	G	7	AAaaAAA	10	GGgGGGGgGG	Yes	No
chrY:4,633,474	C	4	tttT	6	cCCccc	Yes, het	No
chrY:4,939,256	T	13	cCccCcccCCCCC	13	TTTTTTTTTTttT	Yes	No
chrY:4,980,623	T	5	ggggg	7	TtTTTTT	Yes, het	No
chrY:5,355,809^∗^	C	12	TtTTTTTTTtTt	9	cCccccCcC	Yes	Yes
chrY:6,555,594	G	13	TgTttTTtTTtTT	12	GGGGGgGGgGGG	No	
chrY:7,381,330	G	7	cCcCCCc	12	GGGGGgGGgGGG	No	
chrY:12,063,011	C	5	gggGG	8	ccccCCCC	Yes	No
chrY:14,745,277^∗^	A	9	TtTTtTttt	6	aaAaAa	Yes	Yes
chrY:15,126,873	T	7	cccCccc	8	tttTttTT	Yes	No
chrY:15,146,905^∗^	T	4	CCcC	9	tTtTTTTtT	Yes	Yes
chrY:20,627,064	C	9	gGGgGGGG.	5	Ccccc	Yes	No
chrY:27,095,961	T	7	CCcCCCc	8	TTtttTTt	Yes	No
chrY:2,971,542^∗^	A	4	aAAA	14	tTTtTTtttTtttT	Yes	Yes
chrY:4,097,585	C	7	CCcaacc	2	aa	No	
chrY:4,876,956	T	11	aatTTTTTTTT	4	AAAA	No	
chrY:11,970,133	T	10	tttTTTTTTt	6	aaAAaa	No	
chrY:19,883,785	A	5	aAaaA	4	cccc	No	

Second Class							

chrY:13,445,456	G	4	GGGg	1	t	No	
chrY:13,568,272	G	13	aAAgggggggggg	11	aaaAaAaaAAa	No	
chrY:13,833,351	C	17	cCccCCggccCcCcccc	16	CCcCcCCcCttCtttc	No	
chrY:14,573,532	A	21	GAAAAaaAaAAaAaaAAaAAg	5	AAggg	No	
chrY:15,375,202	G	4	GGGg	4	TTTT	No	

An asterisk denotes mutations that were confirmed in blood DNA.

1 Upper case = forward strand; lower case = reverse strand.

## Supplemental Data

Document S1. Two Tables and One Figure

Document S2. Read Depth along the DFNY1-66 Chromosome: .wig file

Document S3. Read Depth along the DFNY1-101 Chromosome: .wig file
