# Supplementary material for: Human Y Chromosome Base-Substitution Mutation Rate Measured by Direct Sequencing in a Deep-Rooting Pedigree
Source: Curr Biol. 2009 Sep 15;19(17):1453–7. doi: 10.1016/j.cub.2009.07.032 (PMC2748900; doi:10.1016/j.cub.2009.07.032)
Supplement: Document S1. Two Tables and One Figure [file mmc1.pdf]

Current Biology Volume *19*

Supplemental Data

Human Y Chromosome Base-Substitution Mutation Rate Measured by Direct Sequencing in a Deep-Rooting Pedigree

Yali Xue, Qiuju Wang, Quan Long, Bee Ling Ng, Harold Swerdlow, John Burton, Carl Skuce, Ruth Taylor, Zahra Abdellah, Yali Zhao, Asan, Daniel G. MacArthur, Michael A. Quail, Nigel P. Carter, Huanming Yang, and Chris Tyler-Smith

Sample Number	M175	M5	M122	PN31	LLY22G	M134	M7	M113	M121	M159	M164	B_DYS389I	B_DYS389II	B_DYS390	B_DYS456	G_DYS19	G_DYS385a	G_DYS385b	G_DYS458	R_DYS437	R_DYS438	R_DYS448	R_Y_GATA_H4	Y_DYS391	Y_DYS392	Y_DYS393	Y_DYS439	Y_DYS635
66	A(1)	G(0)	G(1)	T(0)	C(0)	C(0)	G(0)	T(0)	A(0)	T(0)	A(0)	14	30	23	15	15	12	21	18	14	10	19	11	11	13	12	11	22
101	A(1)	G(0)	G(1)	T(0)	C(0)	C(0)	G(0)	T(0)	A(0)	T(0)	A(0)	14	30	23	15	15	12	21	18	14	10	19	11	11	13	12	11	22

Sample number	DYS472 (B)	DYS508 (G)	DYS487 (Y)	DYS570 (G)	DYS 583(B)	DYS579 (G)	DYS525 (Y)	DYS531 (B)	DYS488 (G)	DYS589 (Y)	DYS578(B)	DYS580(G1)	DYS636(Y)	DYS590(B)	DYS533(G1)	DYS617(Y)	DYS594(G2)	DYS505(B)	DYS641(G)	DYS638(Y)	DYS476(B)	DYS492(G)	DYS540(Y)	DYS537	DYS406S1(G)	DYS568(Y)	DYS480(B)	DYS572(G)
66	8	12	14	18	9	9	10	13	13	13	8	10	10	8	12	16	12	11	7	12	11	12	12	11	11	11	8	12
101	8	12	14	18	9	9	10	13	13	13	8	10	10	8	12	16	12	11	7	12	11	12	12	11	11	11	8	12

Sample number	DYS485(Y)	DYS490(B)	DYS495(G)	DYS567(Y)	DYS494(B)	DYS575(G)	DYS565(Y)	DYS481(G)	DYS576(B)	DYF390S1(G)	DYS569(Y)	DYS618(G)	DYS511(Y)	DYS643(B)	DYS556(G)	DYS573(Y)	DYS530(B)	DYS491(G)	DYS549(Y)	DYS640(G)	DYS554(B)	DYS497(G)
66	10	12	15	10	9	10	11	22	17	3	10	12	11	11	11	10	9	13	12	15	9	11
101	10	12	15	10	9	10	11	22	17	3	10	12	11	11	11	10	9	13	12	15	9	11

Table S1. Y-SNP and Y-STR haplotypes of the DFNY1-66 and DFNY1-101 chromosomes

Primer name	Primer sequence (5'-3')	PCR product (bp)	variants	PCR protocol
DFNY1_101_specific_ 3957219F	TCATTCCAATGGATTTGTGC	541	(G/A)	Touchdown PCR (65ºC to 50ºC, 1ºC decrease/cycle)
DFNY1_101_specific_ 3957219R	AGGTGGATGGATTGCTTGAG			
DFNY1_101_specific_ 4633474F	CTGGAGCTCCCAATTTTTGA	677	(C/T)	general PCR (annealing at 61ºC)
DFNY1_101_specific_ 4633474R	CTTCTGTGGGTTTGGGTTTG			
DNFY1_101_specific_ 4939256F	TGTTGTTCTGCAAAATGTGACTAAT	454	(T/C)	general PCR (annealing at 61ºC)
DNFY1_101_specific_ 4939256R	AACAATTTAGGTATGTGCAATGGAT			
DNFY1_101_specific_ 4980623F	AGTTCCAGTAGTGCCCATGC	509	(T/G)	general PCR (annealing at 61ºC)
DNFY1_101_specific_ 4980623R	TTAGGATAATGGGCAGCAGA			
DFNY1_101_specific_ 5355809F	CCTGCAAGCTCATTCATGGT	678	(C/T)	general PCR (annealing at 61ºC)
DFNY1_101_specific_ 5355809R	CCCACTATGTGACAGGGACA			
DFNY1_101_specific_ 6555594F	TGGAGGATAGGTGCTCTGCT	696	(G/T)	general PCR (annealing at 61ºC)
DFNY1_101_specific_ 6555594R	GGGTCAGTCCCACCTGAATA			
DFNY1_101_specific_ 7381330F	ATGTGCAGCAAGCATGGTAA	519	(A/C)	general PCR (annealing at 61ºC)
DFNY1_101_specific_ 7381330R	GGGACACAGAAAGGCAGAGA			
DFNY1_101_specific_ 12063011F	GTGGCAGACAAGACATGTGG	520	(C/G)	general PCR (annealing at 61ºC)
DFNY1_101_specific_ 12063011R	AATGGGTGAGTCTGGACAGG			
DFNY1_101_specific_14745277	AATTTGGTTGTGTAGGGAGTCAA	599	(A/T)	general PCR (annealing at 61ºC)
DFNY1_101_specific_14745277	GAGTCAGAGTCTCGCTCTTTCAC			

DFNY1_101_specific_15126873F	CAGGTCTAGGAAATGCATACAGG	458	(T/C)	general PCR (annealing at 61ºC)
DFNY1_101_specific_ 5126873R	AGTGAGAAATTGAGCACAGCACT			
DFNY1_101_specific_15146905F	GTTTTGAAACTAAGCAGGTGACG	462	(T/C)	general PCR (annealing at 61ºC)
DFNY1_101_specific_15146905 R	TGGCCATTTAATTTCTAGTTGGA			
DFNY1_101_specific_ 20627064F	ACTGCAAGCTCCGTCTGTCT	645	(C/G)	Touchdown PCR (70ºC to 50ºC, 1ºC decrease/cycle)
DFNY1_101_specific_ 20627064R	TGTGGTGGTAGGTGCCTGTA			
DFNY1_101_specific_ 27095961F	AATACCAACATGGCAATTCAAAC	585	(T/C)	general PCR (annealing at 61ºC)
DFNY1_101_specific_ 27095961R	CTCGTGAGGCTGAGATAGAAGAA			
DFNY1_66_specific_2971542 F	TCTGTTCCTCAGGCTGGAGT	493	(A/T)	Touchdown PCR (65ºC to 50ºC, 1ºC decrease/cycle)
DFNY1_66_specific_2971542 R	GACTCAGTGCGGGTGGTAAT			
DFNY1_66_specific_ 4097585F	GGGTTGGCTAACTGGAATGA	603	(C/A)	general PCR (annealing at 61ºC)
DFNY1_66_specific_ 4097585R	TGATGGACAAATGAAAACCTG			
DFNY1_66_specific_ 4876956F	AATGAGATCACATGGACACAGG	590	(T/A)	general PCR (annealing at 61ºC)
DFNY1_66_specific_ 4876956R	CATCCAAGAGCAATGCTATGAC			
DFNY1_66_specific_ 11970133F	CTTCTCTGGGGAAATCTTTGTTT	583	(A/T)	general PCR (annealing at 61ºC)
DFNY1_66_specific_ 11970133R	GCAAAGGCAGTTTCAATAACATC			
DFNY1_66_specific_ 13445456F	TCCAATTTCCCCAGCAATAG	693	(G/T)	Touchdown PCR (65ºC to 50ºC, 1ºC decrease/cycle)
DFNY1_66_specific_ 13445456R	TTGGATTTGGCAGTGATTTG			
DFNY1_66_specific_ 19883785F	AAGAGCCCCAAAACTTGGTT	613	(A/C)	general PCR (annealing at 61ºC)

DFNY1_66_specific_ 19883785R	AAGGGGTGTTCCCTCTATGG			
DFNY1_66_specific_ 13568272F	TCCAAATCTGCTGATGTGATATG	474	(G/A)	PCR failed
DFNY1_66_specific_ 13568272R	TCTGGCCCAGTAAGATTCAAATA			
DFNY1_66_specific_ 13833351F	CGCACCCAGCTAATTTTTGT	443	(C/T)	general PCR (annealing at 61ºC)
DFNY1_66_specific_ 13833351R	CGCGAGACTCCATCTAAAATAAA			
DFNY1_66_specific_ 14573532F	GTGATGCAGAATATGGGTGATTT	461	(A/G)	general PCR (annealing at 61ºC)
DFNY1_66_specific_ 14573532R	AGTTTGAGCTTCCTGGCTACTTT			
DFNY1_66_specific_ 15375202F	TGATCTTGGCTCACAGCAAC	608	(G/T)	Touchdown PCR (65ºC to 50ºC, 1ºC decrease/cycle)
DFNY1_66_specific_ 15375202R	CGAAAATAGTCCCTGGGTGA			

Table S2. PCR primers used to test candidate mutations using capillary sequencing.

Figure S1. Analysis of non-confirmed candidate mutations by capillary sequencing. The first eight candidate mutations were confirmed in cell line DNA, but not in blood from the cell line donor. The remaining candidates were not confirmed in cell line DNA, and were not tested further. Note that the first mutation, ChrY: 3,957,219 (G→A), lies in an X-Y homologous region and copies from both chromosomes were amplified. '-1' indicates that the non-reference strand is shown.
